# Assessing the potential causal influence of myasthenia gravis on neurodegenerative diseases via multivariable Mendelian randomization

**DOI:** 10.1097/MD.0000000000045340

**Published:** 2025-10-31

**Authors:** Xingwang Huang, Xiuqi Wang, Yi Yang, Hua Chen

**Affiliations:** a Department of Cardiothoracic Surgery, The First Hospital of Nanchang, Nanchang, China; b Department of Cardiovascular Surgery, The Second Affiliated Hospital, Jiangxi Medical College, Nanchang University, Nanchang, China; c Department of Thoracic Surgery, The First Affiliated Hospital, Jiangxi Medical College, Nanchang University, Nanchang, China.

**Keywords:** Alzheimer disease, amyotrophic lateral sclerosis, Mendelian randomization, myasthenia gravis, Parkinson disease

## Abstract

Myasthenia gravis (MG), an autoimmune condition known for impairing neuromuscular signaling, has increasingly been implicated in broader neurological dysfunctions. Recent studies point toward a possible connection between autoimmune and neurodegenerative processes. However, whether MG contributes causally to the onset of major neurodegenerative disorders such as Alzheimer disease (AD), Parkinson disease (PD), and amyotrophic lateral sclerosis (ALS) remains unclear. This study utilizes Mendelian randomization (MR) to explore the potential causal influence of MG on these disorders from a genetic standpoint. A univariable Mendelian randomization (UVMR) framework was employed using summary-level data from genome-wide association studies (GWAS) to evaluate the effect of MG on the risk of AD, PD, and ALS. To confirm the robustness of the association between MG and AD, 2 independent AD GWAS datasets were incorporated for external replication, followed by a meta-analysis to combine the evidence. Additionally, multivariable Mendelian randomization (MVMR) was conducted to adjust for smoking behavior as a potential confounding factor. The UVMR analysis revealed a statistically significant causal relationship between MG and increased susceptibility to AD (odds ratio (OR): 1.037; 95% confidence interval (CI): 1.007–1.068; *P* = .016). No significant causal effects were observed for PD (OR: 1.019; 95% CI: 0.964–1.077; *P* = .509) or ALS (OR: 1.055; 95% CI: 0.977–1.140; *P* = .171). The association between MG and AD was consistently validated in 2 independent datasets (ieu-a-297: OR = 1.084; 95% CI: 1.017–1.156; *P* = .013; ieu-b-2: OR = 1.054; 95% CI: 1.006–1.104; *P* = .027). Meta-analysis reinforced the evidence supporting MG as a risk factor for AD (OR: 1.047; 95% CI: 1.023–1.072; *P* < .001). Furthermore, MVMR adjusting for smoking confirmed that MG independently contributes to AD risk (OR: 1.037; 95% CI: 1.006–1.069; *P* = .020). This study provides robust genetic evidence suggesting that MG is a causal and independent risk factor for AD. These findings highlight a novel link between autoimmunity and neurodegeneration, offering new directions for mechanistic and therapeutic research.

## 1. Introduction

Myasthenia gravis (MG) is a chronic autoimmune neuromuscular disorder characterized by the production of autoantibodies against key components of the neuromuscular junction,^[1,2]^ such as the acetylcholine receptor (AChR)^[3,4]^ and muscle-specific kinase.^[5,6]^ This immune-mediated disruption impairs synaptic transmission, leading to fluctuating muscle weakness and fatigability.^[7,8]^ MG is traditionally considered a disorder confined to the peripheral nervous system, with its primary pathology involving motor function. However, growing evidence suggests that MG may have broader systemic effects, including potential interactions with the central nervous system (CNS).^[9,10]^

Neurodegenerative diseases, including Alzheimer disease (AD), Parkinson disease (PD), and amyotrophic lateral sclerosis (ALS),^[11–13]^ are a diverse group of disorders that are primarily characterized by the progressive dysfunction and eventual loss of neurons, particularly in regions of the brain and spinal cord that are critical for cognitive and motor functions. As these diseases advance, they lead to irreversible impairments in cognition, memory, and movement, severely affecting the quality of life and autonomy of affected individuals. The hallmark features of these diseases are the accumulation of misfolded proteins, such as amyloid plaques in AD,^[14]^ alpha-synuclein aggregates in PD,^[15]^ and TDP-43 inclusions in ALS,^[16]^ which contribute to neuronal toxicity, inflammation, and synaptic dysfunction. Despite extensive research, the exact mechanisms underlying the pathogenesis of these neurodegenerative conditions remain incompletely understood. It is widely recognized that a complex interplay of genetic factors,^[17]^ environmental influences,^[18]^ and aberrant immune responses contribute to the initiation and progression of these diseases.^[19]^ Genetic mutations and polymorphisms in specific genes, such as APOE in AD^[20]^ and LRRK2 in PD,^[21]^ have been identified as major contributors to disease susceptibility. However, environmental factors, such as oxidative stress,^[22]^ toxins,^[23]^ and infections,^[24]^ also play a significant role in triggering or exacerbating the disease processes. These factors often work in tandem to drive the pathological changes observed in neurodegenerative diseases. In addition to genetic and environmental factors, there is an increasing body of evidence supporting the involvement of the immune system in the development and progression of neurodegeneration.^[25,26]^ Chronic inflammation,^[27]^ microglial activation,^[28]^ and dysregulated immune responses^[29]^ have all been implicated in the pathophysiology of these diseases. Microglia, the resident immune cells of the CNS, play a critical role in maintaining homeostasis and responding to injury.^[30]^ However, in neurodegenerative diseases, microglia often become over-activated,^[31]^ leading to the release of pro-inflammatory cytokines and neurotoxic substances that further damage neurons. This chronic inflammatory state can accelerate neurodegeneration and is thought to contribute to disease progression.^[32]^

Given the immunological basis of MG and the involvement of immune processes in neurodegenerative diseases, a potential link between these conditions has been hypothesized. Observational studies have reported an increased risk of cognitive impairment and neuroinflammation in MG patients, particularly regarding AD.^[33–36]^ Several mechanisms have been proposed to explain this association, including systemic inflammation,^[37]^ autoantibody-mediated neuronal damage,^[38]^ and dysfunction of the cholinergic system,^[39]^ which is crucial for cognitive function. However, traditional epidemiological studies are susceptible to confounding factors and reverse causation, making it difficult to establish a definitive causal relationship between MG and neurodegenerative diseases.

Mendelian randomization (MR) offers a powerful approach to addressing these limitations by using genetic variants as instrumental variables (IVs) to infer the causal effects of MG on neurodegenerative diseases.^[40,41]^ Since genetic variants are randomly allocated at conception, MR analysis reduces biases from confounding and reverse causation. Previous MR studies have explored the potential causal association between MG and PD, but these analyses were limited to a single genome-wide association studies (GWAS) dataset.^[42]^ Systematic evaluations of MG in relation to a broader spectrum of neurodegenerative disorders are still lacking. To address this gap, the present study investigates the potential causal role of MG in neurodegeneration, thereby offering new insights into its pathogenic mechanisms and possible implications for intervention.

## 2. Materials and methods

### 2.1. Study design

**Figure 1.**
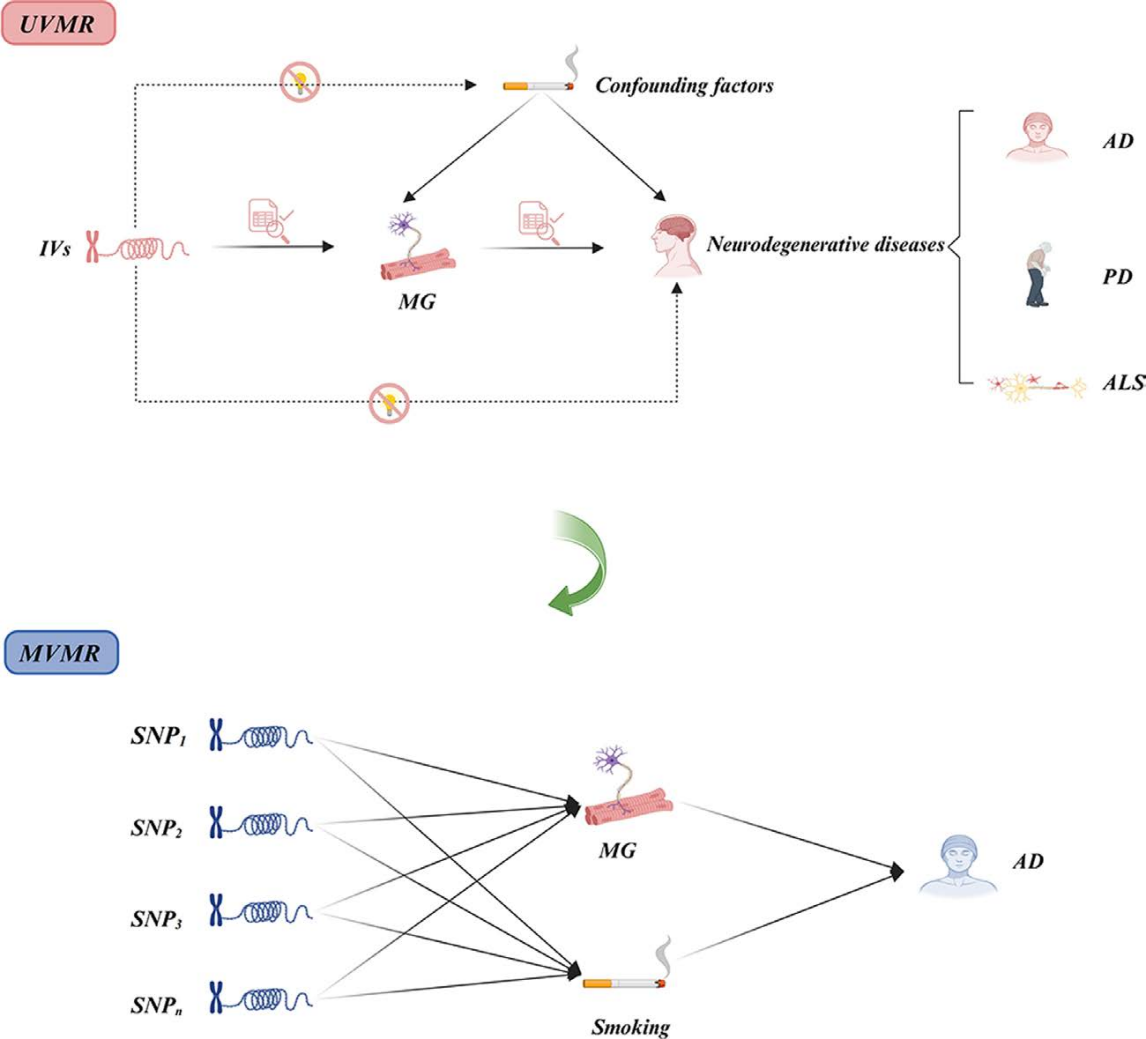
Schematic diagram of UVMR and MVMR. AD = Alzheimer disease, ALS = amyotrophic lateral sclerosis, IVs = instrumental variables, MG = myasthenia gravis, MVMR = multivariable Mendelian randomization, PD = Parkinson disease, SNP = single nucleotide polymorphism, UVMR = univariable multivariable Mendelian randomization.

Based on the core assumptions of MR,^[43]^ univariate Mendelian randomization (UVMR) was initially employed to explore the causal association between MG and neurodegenerative diseases. Additionally, to account for smoking as a potential confounder, multivariable Mendelian randomization (MVMR) was utilized to validate the independent effect of MG on AD (Fig. 1). This study was performed in accordance with the current guidelines for MR analysis.^[44,45]^

### 2.2. Data sources

The GWAS data for MG (ID: ebi-a-GCST90093061, sample size = 38,243) and neurodegenerative diseases, including AD [discovery cohort: ID: ebi-a-GCST90027158, sample size = 487,511; validation cohort: ID: ieu-a-297 (sample size = 54,162) and ieu-b-2 (sample size = 63,926)], PD (ID: ieu-b-7, sample size = 482,730), and ALS (ID: ebi-a-GCST004692, sample size = 36,052), were all obtained from the IEU OpenGWAS database (https://gwas.mrcieu.ac.uk/),^[46]^ which provides a comprehensive platform for accessing large-scale genetic association studies and enables systematic MR analyses. Additionally, the GWAS for smoking (ID: ukb-b-2134, sample size = 424,960), included as a confounding factor, was likewise sourced from IEU. To account for genetic background differences, all participants included in the GWAS datasets were restricted to individuals of European ancestry. As the data were publicly available, no additional ethical approval was required. Detailed information on all GWAS datasets utilized in this MR analysis is summarized in Table 1.

**Table 1:** Detailed information on GWAS data employed in this MR study.

Traits	GWAS ID	Sample size	Case	Sex	Control	Year	Population
MG	ebi-a-GCST90093061	38,243	1873	Males and females	36,370	2022	European
AD (discovery cohort)	ebi-a-GCST90027158	487,511	39,106	Males and females	448,405	2022	European
AD (validation cohort)	ieu-a-297	54,162	17,008	Males and females	37,154	2013	European
AD (validation cohort)	ieu-b-2	63,926	21,982	Males and females	41,944	2019	European
PD	ieu-b-7	482,730	33,674	Males and females	449,056	2019	European
ALS	ebi-a-GCST004692	36,052	12,577	Males and females	23,475	2016	European
Past tobacco smoking	ukb-b-2134	424,960	*NA*	Males and females	NA	2018	European

AD = Alzheimer disease, ALS = amyotrophic lateral sclerosis, GWAS = genome-wide association study, MG = myasthenia gravis, MR = Mendelian randomization, NA = not available, PD = Parkinson disease.

### 2.3. Selection of IVs

The criteria for selecting single nucleotide polymorphisms (SNPs) in this study were as follows: SNPs had to be significantly associated with the MG at the genome-wide level with a threshold of *P* < 5 × 10^−8^, and to ensure independence among SNPs, those in linkage disequilibrium were excluded based on a threshold of *r*^2^ > 0.001 within a 10,000 kb window. Moreover, the minimum allele frequency parameter was set at 0.01, and SNPs with palindromic structures were removed from subsequent analyses. In addition, the *F*-statistic was applied to assess weak instrument bias, meaning that SNPs with *F* < 10 were excluded to avoid potential weak instrument bias, with *F* calculated as *F*=β^2^/SE^2^.^[47]^ Furthermore, SNPs exhibiting horizontal pleiotropy identified by Mendelian randomization pleiotropy residual sum and outlier (MR-PRESSO) were also removed in subsequent analyses to ensure the robustness of the results.^[48]^ Finally, the MR Steiger test was employed to eliminate SNPs with reverse causality,^[49]^ and the remaining SNPs were employed as genetic proxies for MG to analyze its causal effects on neurodegenerative diseases (Table 2).

**Table 2:** Detailed information on SNPs utilized as IVs for MG in this MR study.

SNPs	Effect allele	Other allele	Chromosome number	*F*-statistic
rs2245569	G	A	10	31.81
rs2523596	A	G	6	44.51
rs35274388	A	G	2	30.64
rs4409785	C	T	11	31.99
rs6679677	A	C	1	41.25
rs76815088	C	T	6	58.95
rs4574025	T	C	18	60.66

IVs = instrumental variables, MG = myasthenia gravis, MR = Mendelian randomization, SNPs = single nucleotide Polymorphisms.

### 2.4. MR analysis

For MR analysis, the study employed inverse-variance weighted (IVW),^[50]^ MR-Egger regression,^[51]^ weighted median,^[52]^ and weighted mode methods.^[53]^ The primary analysis was conducted using the IVW method, which assumes all IVs are valid and applies IVW to estimate the causal effect. The IVW method performs regression without considering the intercept term, and the final result represents the weighted average of all IV effect estimates. If the IVW result is statistically significant (*P* < .05), even if other methods do not yield significant results, and no pleiotropy or heterogeneity is detected, the result could still be considered positive as long as the β values from other methods are in the same direction. Notably, in MVMR, least absolute shrinkage and selection operator was additionally introduced as a new method.^[54]^

**Figure 2.**
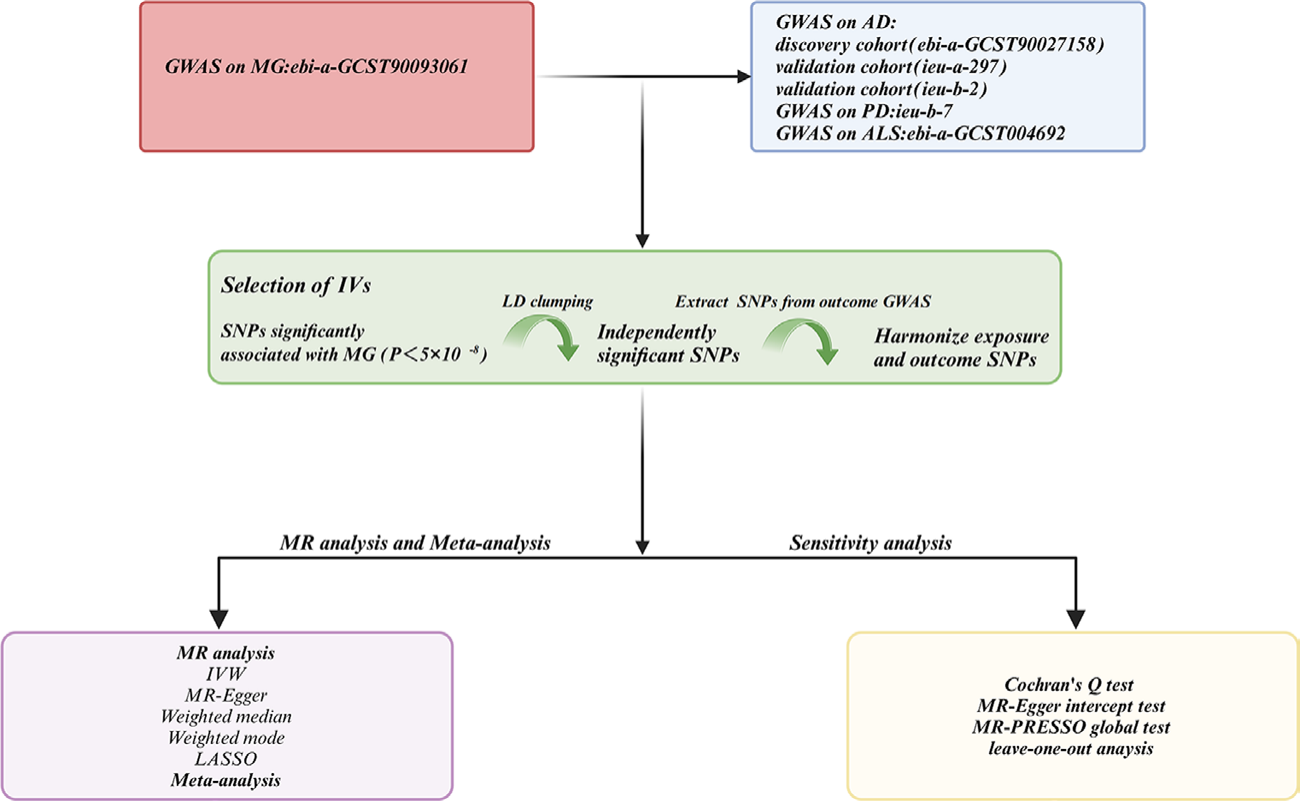
Flowchart of the MR study Investigating the association between MG and neurodegenerative diseases. AD = Alzheimer disease, ALS = amyotrophic lateral sclerosis, GWAS = genome-wide association study, IVs = instrumental variables, IVW = inverse-variance weighted, LASSO = least absolute shrinkage and selection operator, LD = linkage disequilibrium, MG = myasthenia gravis, MR-PRESSO = Mendelian randomization pleiotropy residual sum and outlier, PD = Parkinson disease, SNP = single nucleotide polymorphism.

For sensitivity analyses, Cochran *Q* test was performed to assess heterogeneity among IVs, evaluating differences between them. To detect horizontal pleiotropy across multiple IVs, MR-Egger regression and MR-PRESSO were applied. If the MR-Egger intercept is zero or its *P*-value is not significant, horizontal pleiotropy is considered absent. The MR-PRESSO method identifies pleiotropy by iteratively removing each SNP, recalculating the IVW estimate, and summing the squared residuals of each SNP’s effect relative to the IVW estimate. This process helps determine whether horizontal pleiotropy is significant. Additionally, the leave-one-out method was used by systematically excluding each IV one at a time and recalculating the IVW estimate to assess whether any single IV disproportionately influenced the MR results (Fig. 2).

All statistical analyses in this study were conducted using R software (version 4.1.2). The “TwoSampleMR” package was used for MR analysis, the “MR-PRESSO” package for horizontal pleiotropy testing, and the “ggplot2” package for visualization. A *P*-value of <.05 was considered statistically significant.

## 3. Results

### 3.1. Causal effects of MG on neurodegenerative diseases in UVMR

**Figure 3.**
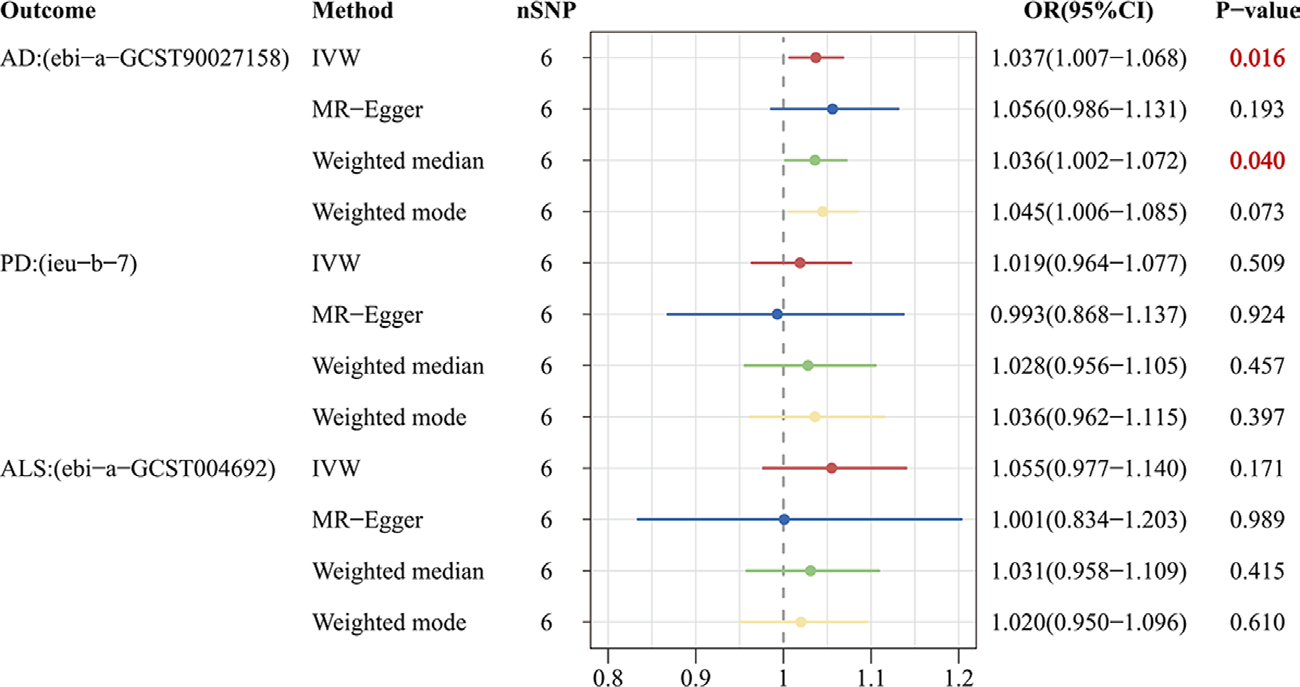
Forest plot presenting the causal effects of MG on neurodegenerative diseases. AD = Alzheimer disease, ALS = amyotrophic lateral sclerosis, CI = confidence interval, IVW = inverse-variance weighted, MG = myasthenia gravis, OR = odds ratio, PD = Parkinson disease, SNP = single nucleotide polymorphism.

**Figure 4.**
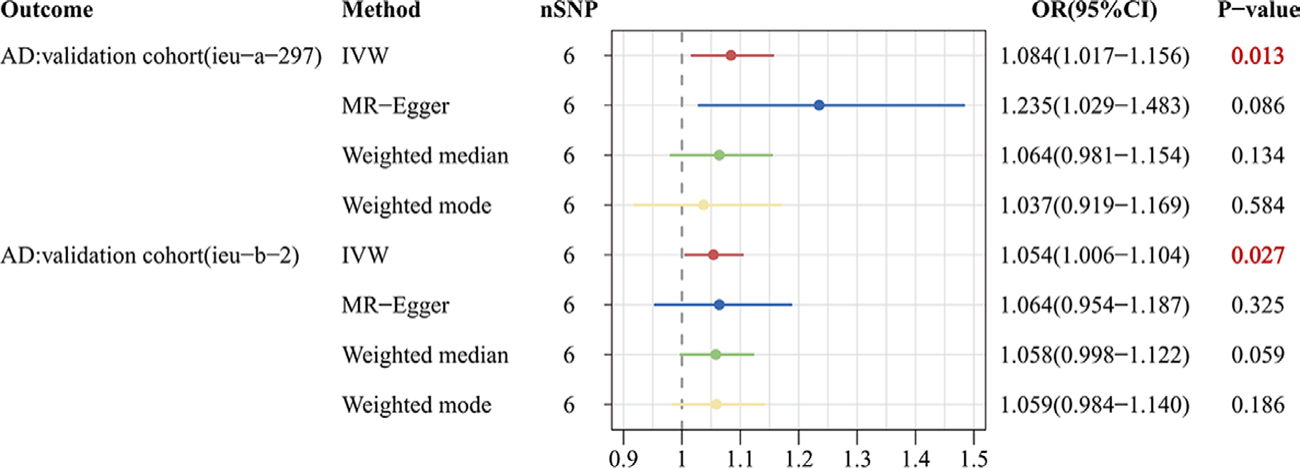
Forest plot presenting the causal effects of MG on AD in validation cohorts. AD = Alzheimer disease, CI = confidence interval, IVW = inverse-variance weighted, MG = myasthenia gravis, OR = odds ratio, SNP = single nucleotide polymorphism.

**Figure 5.**
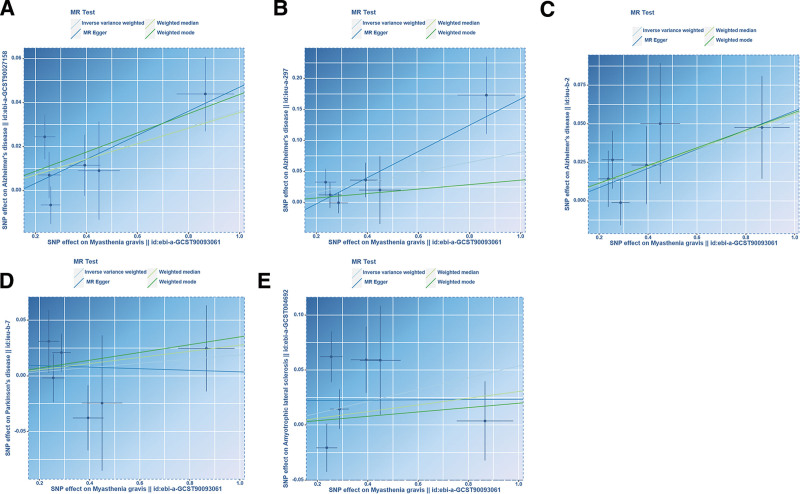
Scatter plots presenting the causal effects of MG on neurodegenerative diseases. (A) Scatter plot presenting the causal effect of MG on AD (ebi-a-GCST90027158) in discovery cohort; (B) Scatter plot presenting the causal effect of MG on AD (ieu-a-297) in validation cohort; (C) Scatter plot presenting the causal effect of MG on AD (ieu-b-2) in validation cohort; (D) Scatter plot presenting the causal effect of MG on PD; (E) Scatter plot presenting the causal effect of MG on ALS. AD = Alzheimer disease, ALS = amyotrophic lateral sclerosis, MG = myasthenia gravis, MR = Mendelian randomization, PD = Parkinson disease, SNP = single nucleotide polymorphism.

In UVMR, the IVW results indicated that MG increased the risk of AD by approximately 3.7% (odds ratio (OR): 1.037; 95% confidence interval (CI): 1.007–1.068; *P* = .016), while no significant association was observed with PD (OR: 1.019; 95% CI: 0.964–1.077; *P* = .509) or ALS (OR: 1.055; 95% CI: 0.977–1.140; *P* = .171) (Figs. 3 and 5). Additional GWAS data on AD also supported this conclusion [validation cohort(ieu-a-297): OR = 1.084; 95% CI: 1.017–1.156; *P* = .013; validation cohort(ieu-b-2): OR = 1.054; 95% CI: 1.006–1.104; *P* = .027] (Figs. 4 and 5).

### 3.2. meta-analysis of the causal effects of MG on AD

**Figure 6.**
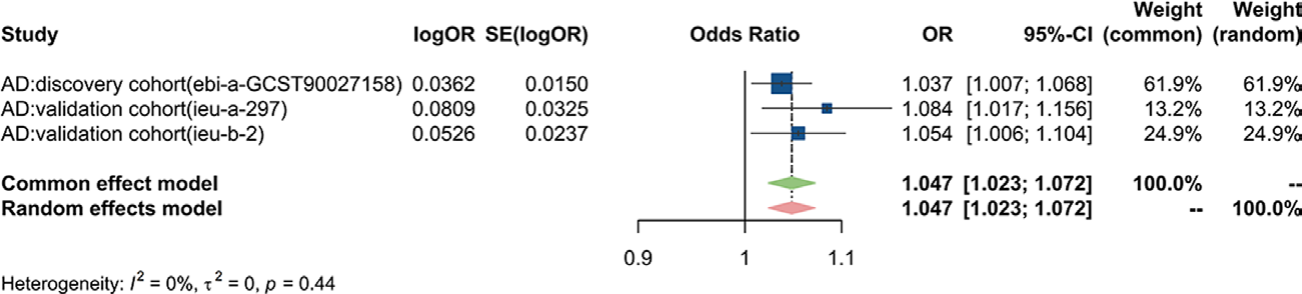
Forest plot presenting the meta-analysis results of the causal effects of MG on AD. AD = Alzheimer disease, CI = confidence interval, MG = myasthenia gravis, OR = odds ratio.

To further validate the reliability of the results, a meta-analysis was conducted to combine effect estimates from different GWAS sources, supporting MG as a potential risk factor for AD (OR: 1.047; 95% CI: 1.023–1.072; *P* < .001) (Fig. 6).

### 3.3. Causal effects of MG on AD in MVMR

**Figure 7.**
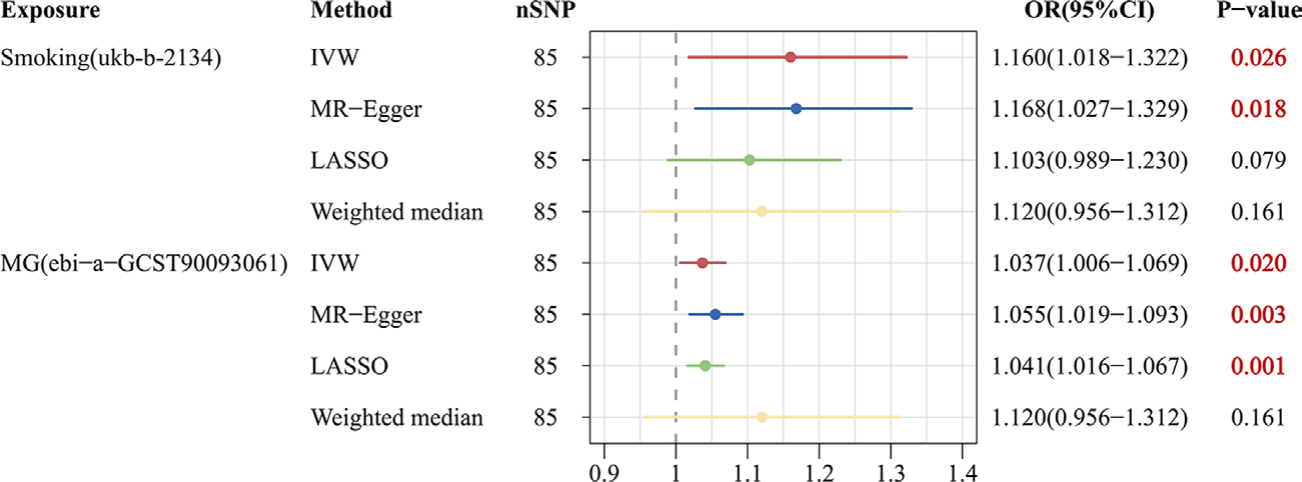
Forest plot presenting the independent causal effect of MG on AD through MVMR. AD = Alzheimer disease, CI = confidence interval, IVW = inverse-variance weighted, LASSO = least absolute shrinkage and selection operator, MG = myasthenia gravis, MVMR = multivariable Mendelian randomization, OR = odds ratio, SNP = single nucleotide polymorphism.

The MVMR results showed that even after accounting for smoking in the MR analysis, the effect of MG on AD remained significant (OR: 1.037; 95% CI: 1.006–1.069; *P* = .020) (Fig. 7), indicating that MG is a potential independent risk factor for AD.

### 3.4. Results of heterogeneity analysis and sensitivity analysis

**Figure 8.**
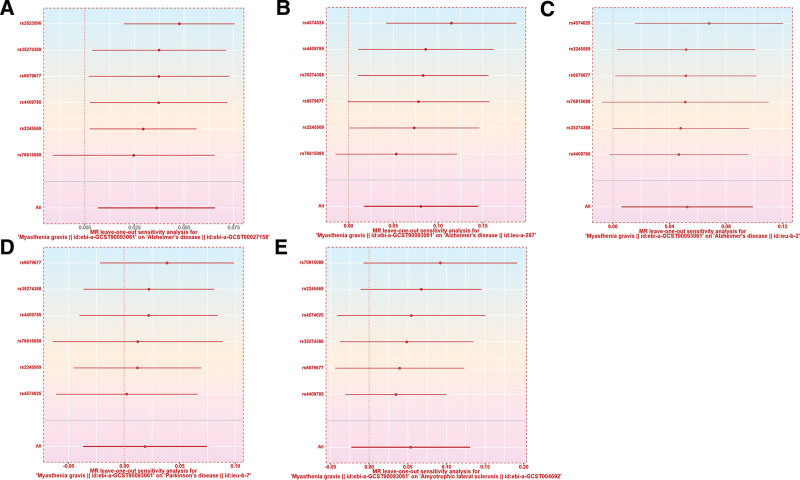
Forest plots presenting the leave-one-out analysis results. (A) Forest plot presenting the leave-one-out analysis result when investigating the causal effect of MG on AD (ebi-a-GCST90027158) in discovery cohort; (B) Forest plot presenting the leave-one-out analysis result when investigating the causal effect of MG on AD (ieu-a-297) in validation cohort; (C) Forest plot presenting the leave-one-out analysis result when investigating the causal effect of MG on AD (ieu-b-2) in validation cohort; (D) Forest plot presenting the leave-one-out analysis result when investigating the causal effect of MG on PD; (E) Forest plot presenting the leave-one-out analysis result when investigating the causal effect of MG on ALS. AD = Alzheimer disease, ALS = amyotrophic lateral sclerosis, MG = myasthenia gravis, MR = Mendelian randomization, PD = Parkinson disease, SNP = single nucleotide polymorphism.

Heterogeneity was evaluated using the Cochran *Q* test, and all results consistently showed no evidence of heterogeneity (*P* > .050). Additionally, the MR-Egger intercept test confirmed the absence of horizontal pleiotropy in the significant results (*P* > .050) (Table 3). Finally, the leave-one-out analysis indicated that the MR results were unlikely to be significantly influenced by any single SNP (Fig. 8).

**Table 3:** Sensitivity analysis results of this MR study.

Outcome	Heterogeneity analysis (Cochran *Q* test)		Horizontal pleiotropy analysis	
	MR-Egger	IVW	MR-Egger intercept test	MR-PRESSO global test
AD (ebi-a-GCST90027158)	0.194	0.251	0.58	0.328
AD (ieu-a-297)	0.521	0.366	0.212	0.354
AD (ieu-b-2)	0.705	0.820	0.856	0.834
PD (ieu-b-7)	0.359	0.473	0.699	0.526
ALS (ebi-a-GCST004692)	0.054	0.069	0.565	0.145

AD = Alzheimer disease, ALS = amyotrophic lateral sclerosis, IVW = inverse-variance weighted, MR = Mendelian randomization, MR-PRESSO = Mendelian randomization pleiotropy residual sum and outlier, PD = Parkinson disease.

## 4. Discussion

This study aimed to explore the causal relationship between MG and neurodegenerative diseases, particularly AD, PD, and ALS through MR approach. While MG has been well-established as an autoimmune neuromuscular disorder primarily affecting the peripheral nervous system, increasing evidence suggests that MG may also have broader systemic effects, potentially interacting with the CNS.^[9]^ The primary objective was to determine whether MG could be a causal risk factor for AD and other neurodegenerative conditions, as well as to examine potential mechanisms that could explain such an association. MR, by using genetic variants as IVs, helps to overcome biases inherent in traditional observational studies, such as confounding and reverse causation, thus providing more reliable insights into causal relationships.

Our findings indicate that MG significantly increases the risk of AD but has no causal association with PD or ALS. This conclusion was based on UVMR analysis, which identified a robust genetic correlation between MG and AD. Further validation using additional GWAS datasets for AD reinforced this relationship, and meta-analysis of these datasets provided further support for the hypothesis that MG could be a potential risk factor for AD. On the other hand, no significant causal link was found between MG and PD or ALS, suggesting that MG’s effect might be specific to certain neurodegenerative diseases, particularly AD. This finding may be explained by differences in disease mechanisms, as AD pathogenesis involves both neuronal degeneration and immune dysregulation.^[55]^ In contrast, PD and ALS are primarily driven by intrinsic neurodegenerative processes with less overlap with autoimmune pathways. In addition, the limited number and strength of MG instruments may have reduced the statistical power to detect modest effects. Given the multifactorial etiology of PD and ALS, MG is unlikely to contribute through a direct causal pathway, although further validation with larger GWAS datasets is warranted.

Several mechanisms could potentially explain the causal link between MG and the increased risk of AD. One plausible pathway is immune dysregulation. MG is characterized by the production of autoantibodies that target key components of the neuromuscular junction, such as acetylcholine receptor and muscle-specific kinase.^[56]^ These autoantibodies lead to impaired neuromuscular function, resulting in fluctuating muscle weakness and fatigue. However, the immune response in MG may extend beyond the peripheral nervous system and influence the CNS.^[57]^ Autoantibodies may cross the blood-brain barrier and interact with neuronal structures, potentially inducing neuroinflammation and contributing to neuronal damage. Chronic inflammation has been increasingly recognized as a key factor in the pathogenesis of neurodegenerative diseases, including AD.^[58]^ In MG, systemic inflammation and activation of immune cells can lead to the release of pro-inflammatory cytokines,^[59–61]^ which may exacerbate neuroinflammation and neuronal injury in the brain.

Another mechanism to consider is the disruption of cholinergic signaling. The cholinergic system is crucial for cognitive functions, including learning and memory, and its dysfunction is a hallmark of AD.^[62]^ MG impairs synaptic transmission at the neuromuscular junction, which could also affect cholinergic signaling in the brain. The disruption of this system in MG could lead to cognitive impairments, potentially contributing to the development of AD. Additionally, neuroinflammation resulting from MG could further damage cholinergic neurons,^[63]^ which are particularly vulnerable in AD. This combined effect of immune dysregulation and cholinergic dysfunction could increase the risk of AD in MG patients.

MR has several advantages over traditional observational studies. One of the major strengths of MR is its ability to mitigate biases from confounding and reverse causation. Since genetic variants are assigned randomly at conception, they are independent of environmental factors or the disease outcomes being studied. This allows MR to provide more accurate estimates of causal effects. In this study, we used genetic variants associated with MG as IVs to infer the causal relationship between MG and neurodegenerative diseases. The use of these genetic proxies provides a robust approach to studying the causal effects of MG on AD, free from the confounding factors that typically affect observational studies.

However, MR also has its limitations.^[64–67]^ One potential issue is horizontal pleiotropy, where the genetic variants utilized as instruments may influence the outcome through pathways other than the exposure of interest. To address this, we performed sensitivity analyses using MR-Egger regression and MR-PRESSO global test to detect and account for pleiotropy. Although these analyses showed consistent results, the possibility of residual pleiotropy cannot be completely ruled out. Another limitation of MR is the reliance on genetic proxies for MG, which may not fully capture the complexity of the disease. MG is a heterogeneous disorder, and genetic variants associated with it may not encompass all the mechanisms contributing to its pathogenesis. Additionally, MG’s effects on neurodegeneration may vary depending on the timing and severity of the disease, which may not be fully reflected in the genetic proxies used. Another limitation of our study is that smoking behavior was the only confounding factor included in the analyses. Although smoking is a well-established risk factor and large-scale GWAS data provide robust instruments for MR, other potential confounders, such as environmental factors and immune dysfunction, could not be comprehensively accounted for due to limitations in publicly available GWAS summary data. Future MR studies incorporating additional confounders would help strengthen the reliability of causal inference. Finally, our study used GWAS data exclusively from European populations, which may limit the generalizability of our findings to other ethnic groups, and further studies in diverse populations are warranted to validate and extend these results.

Despite these limitations, our study provides compelling evidence that MG may be an independent risk factor for AD, highlighting the potential role of immune dysregulation and cholinergic dysfunction in the development of neurodegenerative diseases. The findings underscore the importance of considering MG as a potential contributor to neurodegeneration, particularly in patients with a history of autoimmune disease. Further research, particularly in clinical settings, is needed to explore the underlying mechanisms in more detail and to assess the clinical implications of these findings. This study also emphasizes the value of MR in studying causal relationships, providing a powerful tool to overcome the limitations of traditional observational studies and offering new insights into complex disease interactions.

## 5. Conclusion

In conclusion, our study provides strong evidence that MG may be a causal risk factor for AD, highlighting the potential role of immune dysfunction and cholinergic system disruption. These findings emphasize the need for further research to explore the underlying mechanisms and clinical implications.

## Author contributions

**Conceptualization:** Hua Chen.

**Data curation:** Hua Chen.

**Writing – original draft:** Xingwang Huang.

**Writing – review & editing:** Xingwang Huang, Xiuqi Wang, Yi Yang.
